# A case study of bio-charcoal made from Khat residue for Hawassa City, Ethiopia

**DOI:** 10.1371/journal.pone.0313952

**Published:** 2024-11-20

**Authors:** Endale Fekade Gebreyes, Solomon Tesfamariam Teferi, Kamil Dino Adem

**Affiliations:** 1 Wondo Genet College of Forestry & Natural Resources, Hawassa University, Awasa, Ethiopia; 2 Center for Renewable Energy Addis Ababa Institute of Technology-Addis Ababa University, Addis Ababa, Ethiopia; 3 Addis Ababa Institute of Technology-Addis Ababa University, School of Mechanical and Industrial Engineering, Addis Ababa, Ethiopia; Universiti Teknologi Petronas: Universiti Teknologi PETRONAS, MALAYSIA

## Abstract

The leaves of ’khat’ (*Catha edulis Forsk*), a plant widely grown in Ethiopia are chewed by local people for their stimulant action. Its branches and part of the leaves are thrown as solid waste. The objectives of this study was to characterize fuel briquette made from this waste disposed in Hawassa city, Ethiopia. First, charcoal fines were prepared from the *khat* branches and the leaves. The charcoal fines were then mixed with 20% of clay as a binder. It was followed by molding the mixture in a press machine to produce Branch Charcoal Briquette (BCB) and Leaves Charcoal Briquette (LCB). The experimental results showed the Carbonized Branch Briquette Charcoal (CBBC) has an average calorific values of 19,890 kJ/kg and its other performance parameters are also within the acceptable range. Hence, the city’s *khat* residue can be easily processed into CBBC to substitute yearly 480 ton of charcoal and reduce roughly 1,020 tons of carbon dioxide emission to the environment. The Carbonized Leaves Briquette Charcoal (CLBC) did not gave satisfactory results. Hence, it had been recommended to be studied further.

## Introduction

Almost half of the world’s population and 81% of Sub-Saharan African households depend on wood-based biomass energy resources (particularly firewood, charcoal, agro-residues and dung) for heating and cooking purpose [1]. In Ethiopia, traditional biomass supplies more than 89% of the total energy requirement while the remaining 11% of the energy is supplied from fossil fuel, hydro and other renewable energy sources [2,3]. Reliance on traditional biomass fuels for household energy consumption has high environmental and socioeconomic effects [4].

Charcoal made out of wood is the primary energy source in Sub-Saharan Africa for cooking [5]. Biomass residues have high potential for energy production when properly used [6]. A replacement for charcoal produced from wood could be the use of bio-char for cooking. Bio-char can be produced from various materials, including dried water hyacinth [7], cotton plant residue, and agricultural waste such as sawdust and sugar cane bagasse [8]. Additionally, papyrus wetlands have been identified as a potential source for charcoal briquettes [9]. Khat waste, which has low nitrogen content and no sulphur, is also a viable material for making briquettes used as cooking fuel [10]. Ethiopia, a leading producer, and exporter of khat, ranks third or fourth in importance after coffee, oilseeds, and other agricultural products [11–13].

Khat is a tree that grows at high altitudes throughout the Arabian Peninsula and in the region stretching from eastern to Southern Africa [14]. Many peoples are chewing this to be used as a stimulant. Thereafter, there will be leftovers of Khat categorized as residue. The aim of this research focuses on (i) possible production of bio-char from Khat residue, (ii) study its physical characteristics and (iii) evaluate the performance of bio-char briquettes using the locally available biomass stove named “Merechaye”.

## Methodology

### Raw material collection

The study was conducted in Hawassa city (07°05’ latitude North and 38°29’ longitude East) which is located 273 km south of Addis Ababa at an altitude of 1680 m above sea level. It covers a total area of 157.2 km^2^. It has eight sub-cities, namely: Tabore, Hayek Dar, Menaharia, Misrak, Bahale Adarash, Addis Ketema, Mehale Ketema and Awela Tula. Disposal of khat residue is one of the stinging and widespread problem of the city. The study showed that from the average 6,500 tons of khat consumed every year about 70% is disposed as solid municipal waste [15].

The Hawassa City Administration Revenue Authority provided the estimated data indicating the total volume of Khat supplied to the city from year 2012 to 2016. It was estimated to be in average 6,499,422 kg, with a corresponding estimated wet residue potential of 4,549,595 kg. This shows considerable amount was wasted and disposed as a solid garbage. Table 1 displays the annual taxable consumption of Khat as well as the residual for within these years.

**Table 1:** Hawassa city’s Khat supply and Khat residue [15].

Year	Khat Supply (kg)	Revenue (ETB)	Khat Residue (kg)
2012	4,852,509	24,262,543	3,396,756
2013	5,984,150	29,920,751	4,188,905
2014	6,863,476	34,317,381	4,804,433
2015	7,950,227	39,751,136	5,565,159
2016	6,846,747	34,233,736	4,792,723
Mean ±SD	6,499,422 ± 1,033,038	32,497,109 ± 5,165,189	4,549,595 ±723,126

Experiments were conducted on the wasted branch and leave samples taken from different parts of the city. The khat residue was segregated from other wastes and lastly allowed to dry in open air until its moisture content reached in the range of 12 to 15% as recommended by FAO [16]. The moisture content was determined using the international standard [17].The branches were chopped into small pieces of around 10cm long after being separated from the leaves.

### Carbonization process

**Fig 1:**
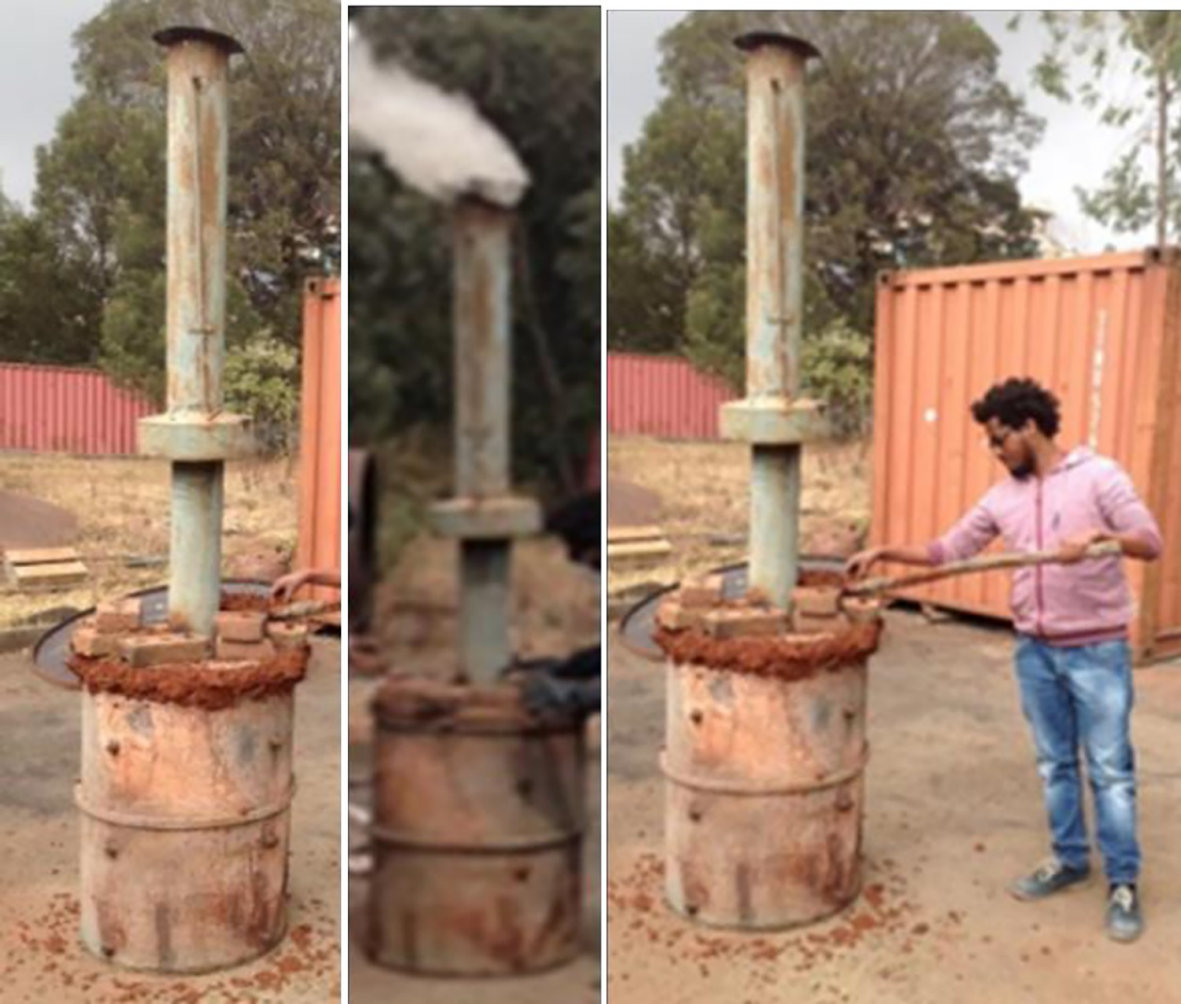
Drum Kiln for carbonization of Khat.

Carbonization is the process of producing energy-rich charcoal by heating biomass to high temperatures in the absence of oxygen. This process increases the energy content of the resulting char [18].The wasted branches and leaves were carbonized inside a ’Drum Kiln’ with a capacity of 200 L [19] fabricated and installed in the workshop of the Ethiopian Ministry of Water, Irrigation and Energy (MoWIE) (Fig 1). The drum kiln was chosen for small-scale carbonization process because of its low cost and simplicity to construct using readily available local materials [20]. To control air infiltration, the bottom part of the kiln was covered by soil, the side was covered by mud and the top was covered by the metal cover.

The color of the flue gases were used to judge the process maturity. At early stage moisture removal is indicated by white smoke followed by yellow smoke to indicate the initiation of the pyrolysis process. Blue smoke indicates that carbonization process is near to be completed [21].

Different factors such as moisture contents of the sample, type of the kiln, air supply, and weather condition can highly affect the carbonization efficiency and the quality of fuel briquette [22]. Conversion efficiency of feed stock (η_fs_) is one of the main indicating parameters used to evaluate effectiveness. It is the ratio of the weight of carbonized feed stoke (m_c.fs_) to the weight of raw feed stock (m_r.fs_) as represented by Eq 1 [23].


$$\eta_{fs}=\frac{m_{c.fs}}{m_{r.fs}}\times 100\%$$ (1)

Proximate analyses were performed on the uncarbonized branches, uncarbonized leaves, carbonized branches and carbonized branches.

### Briquette making and testing

**Fig 2:**
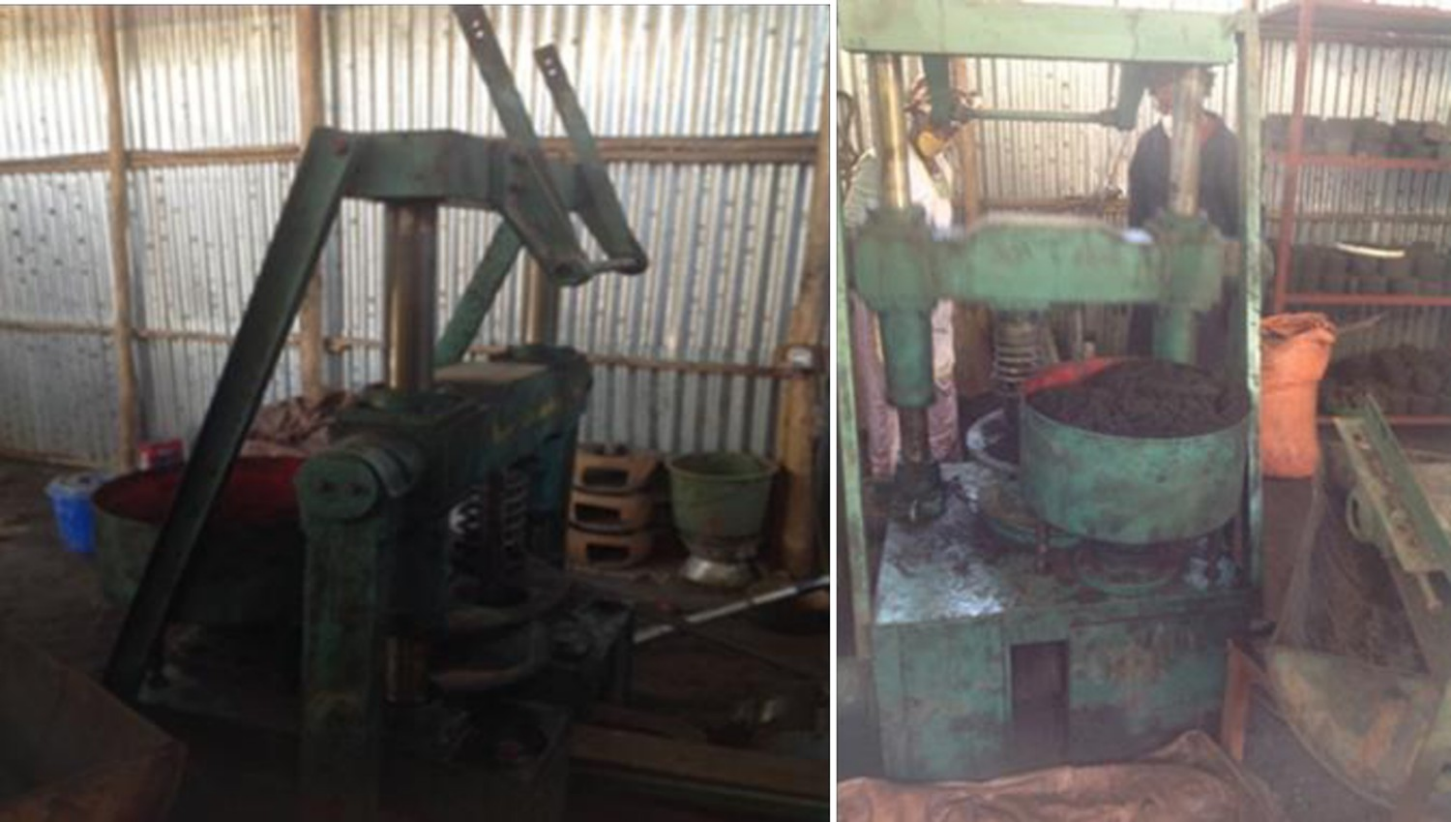
Beehives briquetting machine.

In the preparation of briquette, binding ratio between 10 to 30% have been advised to be the best ratio for quality of the product [24–26]. In this case, 20% of clay was mixed with the carbonized branch charcoal and the carbonized leave charcoal as a binder to get branch charcoal briquette and leave charcoal briquette. Afterward, ’Beehive’ Briquette Machine with press mold was used to get the required shape (Fig 2). The briquettes was kept at compression pressures of 14 MPa to increasing its density and compression strength. Finally, the briquettes were allowed for open air-sun drying [27].

Moisture content, volatile matter content, ash content and fixed carbon content analyses for both briquette charcoals were done following the standard procedure of the American Society for Testing and Materials [28]. Determination of calorific value was also conducted according to [29]. Before experimentation, the instruments were calibrated. At last, the bulk density was determined.

### Water boiling test

**Fig 3:**
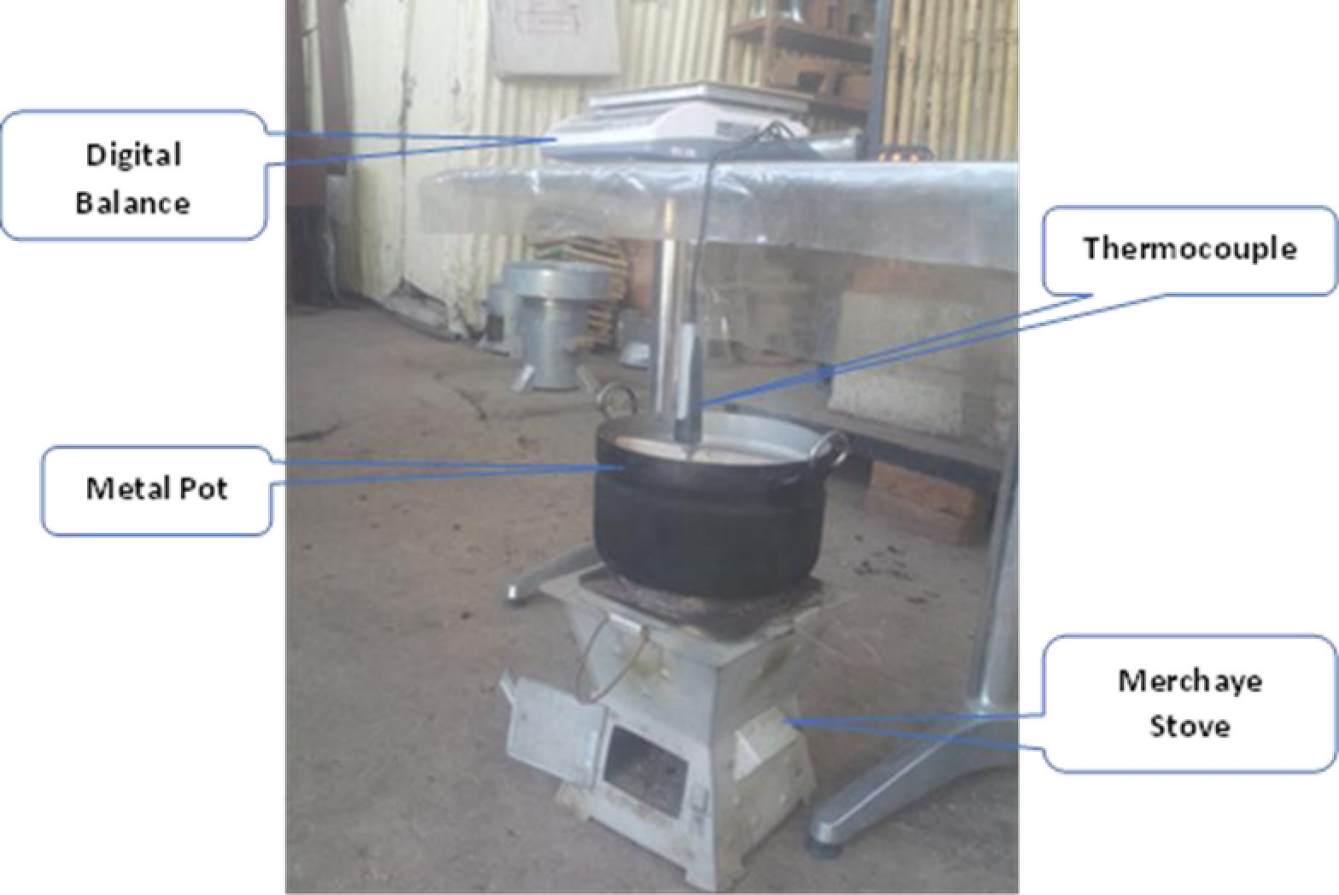
Water boiling test on produced Khat residue briquettes.

Using ’*Merchayae*’ stove, one litter of water was boiled to evaluate the performance of the fuel. The stove has better thermal and fuel saving potential as compared to other traditional charcoal stoves (Fig 3).

### Total emission test

To measure the total Greenhouse Gas Emissions (GHE), the stove was kept inside a closed standard hood. The hood had an overall dimensions of 1000 mm width×750 mm length × 2820 mm height. It has gas mixing chamber with 330–2 LL model multi component gas analyzer. Readings of CO, CO_2_, O_2_, NO, and NO_X_ were taken within 3 minutes interval [30].

### Proximate analysis

#### Moisture content

The crucibles used for determine the moisture content of Khat residue, both as raw material and after carbonization and briquette production, were first preheated in a muffle furnace at 750°C for 10 minutes. After preheating, the crucibles were cooled in a desiccator for 1 hour. They were then weighed, and 1g of sample was added to each crucible using a balance with a precision of 0.1 mg. Following this, the samples were placed in an oven at 105°C for 2 hours. After drying, the samples were transferred to a desiccator for 1 hour before recording their weights. The process was repeated until a constant mass was achieved. The same specimen was also used for determining the volatile and ash content. Then, the moisture content was calculated by using Eq 2 [28].

Moisture,%=[(A–B)/A]×100 (2)

Where: A = grams of air-dry sample used, and

B = grams of sample after drying at 105°C.

#### Volatile content

The volatile matter content of Khat residue raw material, after carbonization and briquettes were determined by preheating the crucibles used for the moisture determination at 950°C by preheating the crucibles, covers and samples in the muffle furnace door open, for two minute on the outer edge of the furnace at 300°C, then heating for three minutes on the edge of the furnace at 500°C, then move the samples to the rear of the furnace for six minutes with the muffle furnace door closed at 950°C in covered crucible of specimen by lid or metal box prepared for this purpose. Finally, cool the samples in a desiccator for one hour and weigh. The volatile content was determined using Eq 3 [28].

Volatilematter,%=[(B–C)/A]×100 (3)

Where,

C = grams of sample after drying at 950°C

#### Ash content

In order to determine the ash content, place the lids and the uncovered crucible used for the volatile matter determination, and containing the sample in the muffle furnace at 750°C for 6 hour. Finally, the crucibles were cool with lids in placed in a desiccator for 1 hour and then, the weight is a noted. The ash content was calculated as the proportion of grams of residue to grams of sample after drying at 105°C using Eq 4 [28].

Ash,%=(D/B)×100 (4)

Where,

D = grams of residue

#### Fixed carbon

The percentage of carbon present in a particular sample is mentioning to carbon content. During combustion, the percentage of available carbon is the fixed carbon of fuel that is not equal to the total amount of carbon because there is also a significant amount that was released as hydrocarbons in the volatiles. The percentage of fixed carbon content of the sample in this study was computed using Eq 5 [28].


FC%=100‐(MC%+VM%+Ash%) (5)

#### Calorific value

To measure the calorific value, a Par 6200 Calorimeter with a standard 1108 Oxygen Bomb was used at the Alternative Energy Development and Promotion Directorate Laboratory in Ethiopia. The briquette samples were analyzed using an adiabatic oxygen bomb calorimeter, specifically the Parr 6200 model, with oxygen bomb models Parr M39889 and Parr M39805, following the Par instruction manual as per standards ASTM [31]. Similarly, the briquette samples, weighing between 0.7–1 grams, were pulverized, placed in a capsule, combusted, and the calorific value was measured using the calorimeter.

#### Bulk density

The bulk density (BD) of the briquettes was determined by selecting 10 briquettes at random. Their weights were measured using a weighing balance, while the volume of each briquette was calculated by averaging the diameters and heights measured at two different positions using a Vernier caliper. For the beehive briquettes, the volume was adjusted by subtracting the volume of the 12 holes from the total volume to obtain the actual volume of the briquettes [32]. BD was calculated by using Eq 6 [33].


BD(g/cc)=(massofbriquettesample)/(volumeofbriquettesample) (6)

## Result and discussion

After conducting test on selected 10 khat shops and chewers taken from 1kg khat, 0.70 kg khat residue were found (Table 2). This result confirms the assessment done by Hawassa City Adminstration [15].

**Table 2:** Amount of khat residues from one kilogram (1 kg) khat.

Trial No	Amount of Khat (kg)	Amount of Khat residues (kg)
1	1.00	0.70
2	1.00	0.80
3	1.00	0.79
4	1.00	0.69
5	1.00	0.65
6	1.00	0.62
7	1.00	0.63
8	1.00	0.72
9	1.00	0.65
10	1.00	0.76
Mean ± SD	1.00	0.70 ± 0.06

The kiln was preheated until white smoke came up from the chimney that denotes sample dehydration. An opening at the bottom of the kiln was left open until the fire reached 250°C, at which point the door chimney was shut. The chimney and all other openings were sealed to limit the amount of oxygen present and to create an atmosphere that was ideal for carbonization process to take place above 300°C. After that, the carbonization process was carried out continuously in the absence of air for two hours. Carbonization process was performed on well cleaned and dried 15 kg of khat branches residue and 15 kg of khat leaves residue. As a result, 4.25 kg of carbonized branches charcoal (CBC) and 4.18 kg of carbonized leaves charcoal (CLC) were produced. They had conversion efficiencies of 28.33% and 27.89%, respectively. The variation is not so significant as compared to the 30% conversion efficiency reported by Seboka [34]. The dried khat residue goes through a pyrolysis process when it is carbonized. As a result, the complex organic molecules in the khat residue were broken down into simpler substances, primarily the solid carbon-rich material (charcoal) and several volatile compounds, including water vapor, carbon dioxide, and methane. The resulting solid charcoal will weigh significantly less than the initial weight of the dried khat residue since these volatile compounds have left the solid.

CBC and CLC were further crushed into powder and separately mixed with 20% of clay. ’Beehive’ Briquette Machine" was then used to mold the charcoal mud into briquettes. The wet briquette charcoals were sun dried and gave 5.10 kg of dried Carbonized Branch Briquette Charcoal (CBBC) and 5.02 kg of Carbonized Leaves Briquette Charcoal (CLBC).

The results of proximate analysis listed in Table 3 showed that the Moisture Content (MC) of both raw khat branches and leaves significantly decreased after canonization. The MC of the carbonized branch was 2.67% which raised to 3.33% after being converted into briquette. Similarly, the MC of carbonized leaves was raised from 3.2% to 4.0%.The raise in MC is mainly due to the high porosity (water adsorbing capacity) of the clay added in both fuels as a binder. However, it is still less as compared to that of the briquette made from cashew nut shell having a MC between 5 to 6% [20]. Other studies also showed that the MC of briquette made from rice husk and corncob briquettes were 12.67% and 13.47%, respectively [35]. For smooth heat transfer, FAO recommended MC not to exceed 15% [36]. A lower moisture content of briquettes is one of the indicators showing to have higher calorific value [37].

**Table 3:** Proximate analysis and physical properties of raw, carbonized and briquettes khat.

Samples	Average Values of the Proximate Analysis				Calorific Value	
	MC (%)	VM (%)	AC (%)	FC (%)	BD (kg/m^3^)	CV (kJ/kg)
**Raw khat**						
Raw khat branches	10.85	70	4.35	14.8	-	15,851
Raw khat leaves	11.48	71	7.23	10.29	-	17,204
**Carbonized khat**						
CBC	2.67	18.85	3.48	75.00	-	24,863
CLC	3.20	26.57	5.79	64.44	-	17,295
**Carbonized khat Briquette charcoals**						
CBBC	3.33	23.57	23.62	49.48	730	19,890
CLBC	4.00	33.22	26.62	36.17	710	13,836

The Volatile Matter contents (VM) of CBBC and CLBC were 23.57% and 33.22%, respectively which were found to be within the acceptable range of 5 to 40% [38].

The test results revealed that the Fixed Carbon (FC) content decreased from 75% to 49.48% in the CBC and from 64.44% to 36.17% in the CLC. This reduction in FC for the charcoal briquettes is attributed to the increase in Ash Content (AC), caused by the addition of clay as a binder.

The fixed carbon content of charcoal briquette produced from mixed tropical hardwood was between 68.6% and 69.8% [38]. This valued for Neem wood residue bonded with gum Arabic and starch was 84.38% and 84.31%, respectively [39]. Charcoal briquette from sesame stalk and a mixture of carbonized branch powder with carbonized leaves powder had 44.4% FC as reported by Gebresas, Asmelash [24]. In case of charcoal briquette made from sawdust, FC content even went lower, about 20.7% [37]. Hence, The FC content of the briquette charcoals from khat residues had reasonably good values.

Charcoal briquette with less Ash Content (AC) are preferred since their combustion efficiencies were higher [37]. A good quality wood charcoal have an Ash Content (AC) usually fluctuating between 3 to 4%. The test results showed that CBC with AC 3.48% fulfilled good quality charcoal since it is within the range. However, CLC which had 5.79% AC failed to fulfill this quality. The raise in AC contents of briquettes charcoal were due to the availability of clay.

The Bulk Densities (BDs) of CBBC and CLB C were 730 kg/m^3^ and 710 kg/m^3^, respectively for. These were higher than that of Eupatorium briquette with bulk density 330 kg/m^3^ [40]. However, BDs were less than the values for charcoal briquette produced from coconut husks, sawdust and banana leaves which were 760 kg/m^3^, 890 kg/m^3^ and (990 to 1000) kg/m^3^,respectively [41,42].

The gross Calorific Value (CV) of CBBC was 19, 890 kJ/kg.K. This is higher that the CV of CLBC which was 13, 836 kJ/kg.K. Nitrogen concentrations varied significantly across plant organs, with larger concentrations detected in the leaves, roots, branches, and trunk respectively. Leaf nitrogen is 2.7 times higher than branches that potentially affected the calorific value [43]. The gross calorific value of charcoal briquette made from coffee pulp was about 16,905 kJ/kg whereas the gross calorific value of charcoal briquette made from coffee husk was around 21,106 kJ/kg [44]. The calorific value of wood was reported to be roughly 13,803 kJ/kg [45]. This showed that both briquette charcoals of this study had acceptable gross calorific value.

Few seconds after the water boiling test was started, the combustion process of CBBC was observed having neither smoke, smell, sparks nor soot. However, the combustion of CLBC was accompanied by observable smoke and smell even 20 min after the startup.

**Fig 4:**
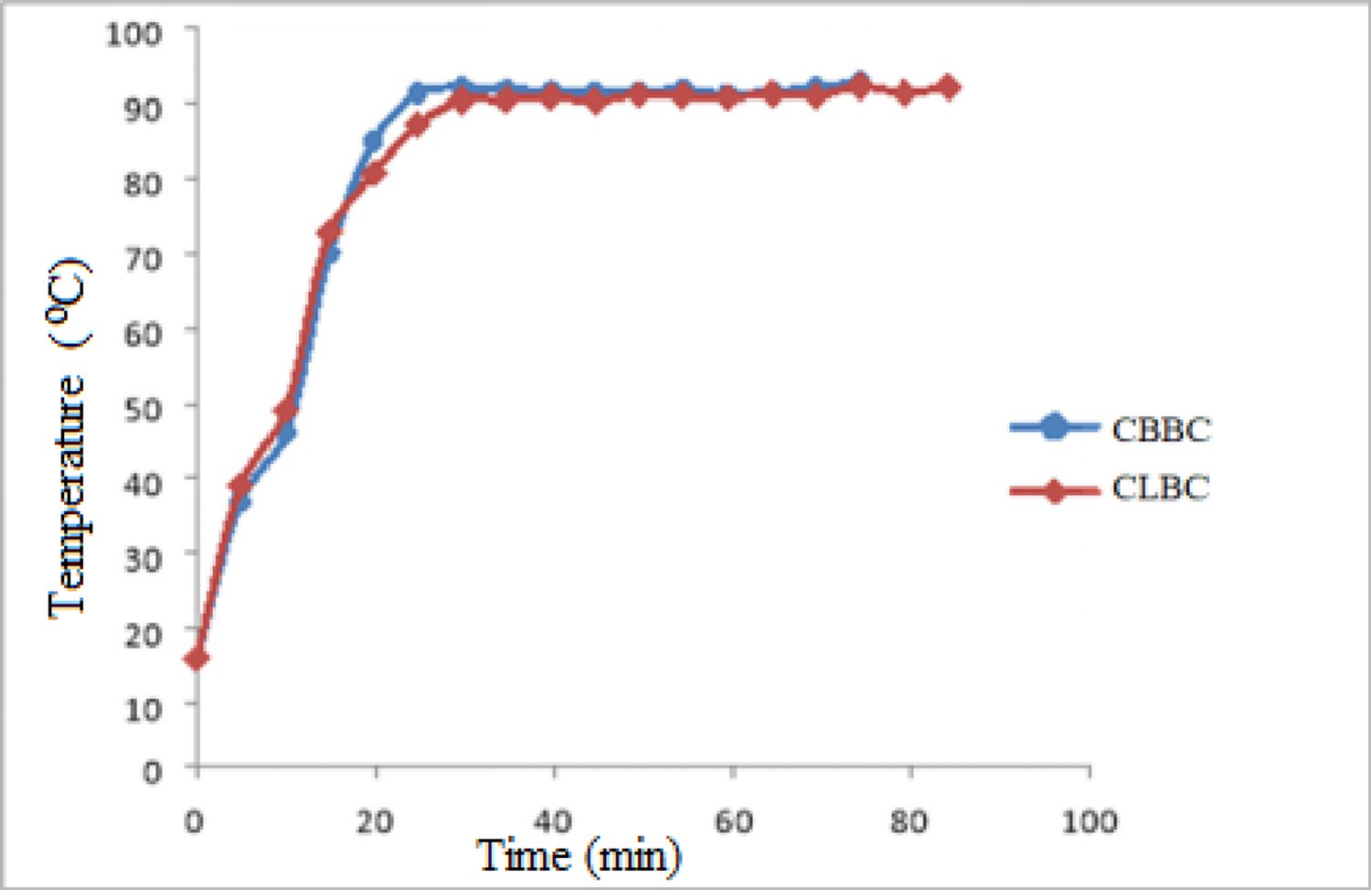
Temperature versus time curve to boil one liter of water.

The time spent to boil 1 litter of water is illustrated in Fig 4. Time taken to start boiling one liter of water using CBBC was 23 min which is near to the time taken when sesame stalk was employed to boil water in 20 minutes [24]. The time taken to start boiling the same amount of water using CLBC was 35 min.

The average time taken for a complete conversion of the entire charcoal into ash was 71 min and 82 min respectively for CBBC and CLBC, respectively.

Emission test

Table 4 shows a comparison between the total gas emissions of khat residue fuel briquette with the recommended preferable range of CO, CO_2,_and O_2_. The table clearly indicated that the gas emission produced by the fuel briquettes confirms to the recommended range of Indian standard for portable solid biomass cook stove [30].

**Table 4:** Selected toxic emissions with the maximum exposure limit.

Emissions (avg)	CO_2_ (%)	CO (ppm)	O_2_ (%)	NO (%)	NO_X_ (%)
CBBC(500 g)	0.37	728	20.20	2.27	0
CLBC(500 g)	0.48	831	20.08	7.21	0
Preferable standard range	0–20	0–1000	0–25	-	-

Where 1% CO = 10,000 ppm.

CBBC and CLBC had total CO emissions of 728 ppm (0.07%) and 831ppm (0.08%), respectively. They had lower CO emission than charcoal briquettes produced from banana peel and banana bunch which had total CO emission of 3463 ppm (0.35%) and 1568 ppm (0.16%), respectively as reported by Mopoung and Udeye [46]. According to international standard for the Determination of Toxicity of Gases [46], CO will not be considered as toxic if its concentration in the atmospheric air does not exceed 4,947 ppm (0.5%). Hence, both briquettes do not cause any threat to consumers. However, a person near the stove felt headache, nausea and dizziness 45 minutes after CLBC started to burn. This may be due to the higher concentration of NO in the fuel. Hence, it is recommended comfortably to use branches of khat for the production of briquette charcoal while khat’s leave residue needs further study to be used as a fuel source or for other purposes like production of fertilizer.

Moreover, the thermal performance of CBBC was in the acceptable range for all other parameters (moisture content, volatile matter content, ash content, fixed carbon bulk density and calorific value) as it has been compared with other research work and discussed earlier. On the other hand, the test results showed CLBC didn’t thermally perform well for some of these parameters.

### Charcoal recovery potential

Totally, 200 kg of wet khat residue was randomly collected from different sites of the city. After being allowed to dry in an open air, 120 kg (62 kg-branch and 58 kg-leaves) of dried khat which is 60 percent of the wet one was remained. To estimate the charcoal production potential from the khat inflow, five years data (from 2012 to 2016) were collected. The data showed the average annual khat in flow to be around 6500 tons, Table 5.

**Table 5:** Yearly charcoal recovery potential of Hawassa city.

Material	Yearly average value (t/year)	
khat inflow		6,499.4
Wet khat residue (70% of khat inflow)		4,549.6
Air-dry khat residue (60% of Wet khat residue)		2,729.6
Air-dry khat branch residue (51.67% of Air-dry khat residue)		1,410.4
Total CBC production potential (28.33% of Air-dry khat branch residue)		399.6
Total CBBC production potential (Total CBC+20% Ash as a binder)		479.5

People chew about 30 percent of the raw khat and throw the remaining 70 percent as residue which lead the city to have about 4550 tons of khat as a solid waste per annum. Accordingly, the city had a yearly potential of producing around 400 tons of CBC or 480 tons of CBBC from khat residue.

Since, CBBC has a calorific value of 19890 kJ/kg, it can provide approximately 9.54 billion kJ of energy per annum. This substitutes nearly 309 tons of charcoal or save 1855 m^3^ of fire wood from burning since the specific energy content of charcoal is nearly 30,800 kJ/kg and one ton of charcoal can be obtained from 6 m^3^ of fuel wood [45].

According to [Girard [47]], 250 kg of charcoal have 225 kg of carbon within it and 300 kg of carbon is equivalent to 1.1 tone of CO_2_ which means 250 kg of charcoal is equal to 0.825 tons of CO_2_. Therefore, the production of fuel briquettes from carbonized khat branch could possibly reduce 1,020 tons of CO_2_ emission every year.

## Conclusion

The use of charcoal briquettes made from khat residue offers a promising alternative to traditional wood-based charcoal, which is often produced through inefficient and environmentally harmful methods. This study found that fuel briquettes derived from khat residue are a high-quality energy source and could serve as an effective waste management strategy. This approach has the potential to significantly mitigate deforestation by providing an alternative fuel source. According to this study, nearly 480 tons of khat residue-based charcoal briquettes (CBBC) could be produced annually, substituting 309 tons of traditional charcoal and reducing approximately 1,020 tons of CO_2_ emissions. To further support this sustainable practice, it is recommended that governments in developing countries, such as Ethiopia, increase investment in briquette production to lessen the reliance on firewood.

## Supporting information

S1 File (PDF)
