# Supplementary material for: A case study of bio-charcoal made from Khat residue for Hawassa City, Ethiopia
Source: PLoS One. 2024 Nov 20;19(11):e0313952. doi: 10.1371/journal.pone.0313952 (PMC11578448; doi:10.1371/journal.pone.0313952)
Supplement: S1 File — (PDF) [file pone.0313952.s001.pdf]

Supporting Information

Table 1: The proximate analysis and physical properties of briquettes made from carbonized Khat residue with clay soil binder (1:5 ratios).

Samples	Treat-ments	Proximate analysis (%)				Physical property	
		MC in %	VM in %	AC in %	FC in %	BD in g/cm^3	CV in kJ/kg
Briquettes made from carbonized branch residue	T_1	4.00	22.70	25.26	48.04	0.67	19,140.46
	T_2	3.00	24.80	19.28	52.92	0.77	19,619.68
	T_3	3.00	23.20	26.33	47.47	0.75	20,911.81
	Mean ±SD	3.33 ± 0.47	23.57 ± 0.90	23.62 ± 3.10	49.48 ± 2.45	0.73 ± 0.04	19,890.65± 748.10
Briquette made from carbonized leaves residue	T_1	3.86	33.34	28.38	34.28	0.69	13,849.43
	T_2	4.22	31.26	27.67	37.07	0.73	12,611.31
	T_3	3.92	35.05	23.80	37.15	0.71	15,049.33
	Mean ±SD	4.00 ± 0.16	33.22 ± 1.55	26.62 ± 2.01	36.17 ± 1.33	0.71 ± 0.02	13,836.70± 995.36
Briquette made from carbonized branch and leaves	T_1	3.44	29.74	23.07	43.75	0.65	16,934.78
	T_2	3.69	28.96	25.01	42.34	0.76	16,039.02
	T_3	3.82	25.92	27.10	43.16	0.76	17,980.46
	Mean ±SD	3.65 ± 0.16	28.21 ± 1.65	25.06 ±1.65	43.08± 0.58	0.72± 0.05	16,984.75± 793.38
Briquette made from carbonized branch and un-carbonized leaves	T_1	9.26	57.78	28.24	4.72	0.48	2,000.62
	T_2	9.19	59.29	24.93	6.59	0.44	2,057.24
	T_3	9.90	59.87	27.12	3.11	0.53	1,906.96
	Mean ±SD	9.45± 0.32	58.98± 0.88	26.76± 1.37	4.81± 1.42	0.48± 0.04	1,988.27± 61.97

Table 2: Relative time taken to boil one litter of water; done using Merchaye-stove

Briquette type	Average time taken to boil one litter Water in Minutes	Average time taken to change the water into vapor in (Hour & Minutes)	Average time taken to turn to Ash (Hour & Minutes)	Average Calorific Value (kJ/kg)
Carbonized Khat branch residue briquette	23<br>22<br>24	1hr and 9 Min<br>1hr and 12 Min<br>1hr and 11 Min	2hr and 46 Min<br>2hr and 48 Min<br>2hr and 50 Min	19,140.46<br>19,619.68<br>20,911.81
Mean ± SD	23 ± 1.00	1hr and 11Min± 1.53	2hr and 48 Min ± 2.00	19,890.65 ± 565.88
Carbonized Khat leaves residue briquette	36<br>33<br>37	1hr and 19 Min<br>1hr and 24 Min<br>1hr and 22 Min	2hr and 14 Min<br>2hr and 10 Min<br>2hr and 19 Min	13,849.43<br>12,611.31<br>15,049.33
Mean ± SD	35± 2.12	1hr and 22 Min ± 2.52	2hr and 14 Min ± 4.51	13,836.70± 1219.07

በኢትዮጵያ ፌዴራላዊ ዲሞክራሲያዊ ሪፐብሊክ
የውሃ ፣ መስኖና ኤሌክትሪክ ሚኒስቴር
The Federal Democratic Republic of Ethiopia
Ministry of Water, Irrigation & Electricity

ቁጥር ________________
Ref.No MaWIE 260/11/221
08 MAY 2018

To: Hawassa University Wondo Genet College of Forestry and Natural Resources
Hawassa

Subject: **Letter of certification and experimental results**

As stated in the subject Hawassa University Wondo Genet College of Forestry and Natural Resources asked our ministry with Ref.No 1562/1.23/17 and on date 12 Dec 2017 for Mr. Endale Fekadu Gebreyes to do laboratory work on his thesis research paper, entitled "**Production and Characterization of Fuel Briquette from Khat (*Catha EdulisForsk*) Residue for Diversification of Household Energy Sources**."

Therefore, it is to inform you that the aforementioned M.Sc. student has done properly his experimental work and has obtained his results in Ministry of Water, Irrigation and Electricity in Alternative Energy Development and Promotion Directorate Laboratory and Workshop section, located at Gurd Sholla Addis Ababa, with the coordination of the workshop experts, from January to April 2018 and for assurance a 3 page laboratory results are attached with this paper.

With Regards

Asress Wolde-Giorgis
Alternative Energy Technology Development & Promotion Dire[illegible]
Director

የኢትዮጵያ ፌዴራላዊ ዲሞክራሲያዊ ሪፐብሊክ የውሃ ፣ መስኖና ኤሌክትሪክ ሚኒስቴር 1
The Federal Democratic Republic of Ethiopia
Ministry of Water, Irrigation & Electricity

እባክዎን መልሱን በሚጽፉልን ጊዜ የእኛን ደብዳቤ ቁጥር ይጥቀሱልን
Please Quote our Ref. No. When Replying

ስልክ 011-6-61-11-11
Tel. 011-6-63-72-22

ቴሌ ፋክስ 011 - 6-61-08-85
Telefax 011 - 6-61-07-10
011 - 6-62-73-69

ፖ.ሣ.ቁ. 5744
P.O.Box 5673

አዲስ አበባ - ኢትዮጵያ
ADDIS ABABA - ETHIOPIA

Table 1.1 Laboratory experimental results of proximate analysis of raw Khat branch and leave residues

Samples	Trial	Proximate analysis (%)			
		MC in %	VM in %	AC in %	FC in %
RawKhat branch	1	10.67	71.00	4.46	13.87
	2	11.33	68.67	4.10	15.90
	3	10.56	70.33	4.48	14.63
RawKhat leaves	1	11.44	73.00	7.77	7.79
	2	11.67	70.00	7.14	11.19
	3	11.33	70.00	6.79	11.88

Table 1.2 Laboratory experimental results of proximate analysis carbonized Khat branch and leaves residues

Samples	Trial	Proximate analysis (%)			
		MC in %	VM in %	AC in %	FC in %
Carbonized Khat branch residue	1	3.20	18.16	3.57	75.07
	2	2.40	19.84	3.28	74.48
	3	2.40	18.56	3.58	75.46
Carbonized Khat leaves residue	1	3.09	26.67	6.22	64.02
	2	3.38	25.01	5.71	65.90
	3	3.14	28.04	5.43	63.39

Table 1.3 Laboratory experimental results of proximate analysis of briquettes with clay soil binder (1:5 ratios)

Samples	Trial	Proximate analysis (%)			
		MC in %	VM in %	AC in %	FC in %
Briquettes made from Khat branch	1	4.00	22.70	25.26	48.04
	2	3.00	24.80	19.28	52.92
	3	3.00	23.20	26.33	47.47
Briquettes made from Khat leaves	1	3.86	33.34	28.38	34.28
	2	4.22	31.26	27.67	37.07
	3	3.92	35.05	23.80	37.15
Briquette made from carbonized branch and leaves (52%: 48%)	1	3.44	29.74	23.07	43.75
	2	3.69	28.96	25.01	42.34
	3	3.82	25.92	27.10	43.16
Briquette made from carbonized branch with un-carbonized leaves (23%:77%)	1	9.26	57.78	28.24	4.72
	2	9.19	59.29	24.93	6.59
	3	9.90	59.87	27.12	3.11

Archive
Ministry of Water, Irrigation & Electricity

Table 1.4 Laboratory experimental results ofgross calorific values of raw, carbonized and briquettes of Khat branch and leaves residue

Samples	Trial 1 (kJ/kg)	Trial 2 (kJ/kg)	Trial 3 (kJ/kg)
Raw Khat branch	16,043.82	15,633.51	15,876.35
Raw Khat leaves	16,880.26	17,469.34	17,263.47
Carbonized Khat branch	23,925.58	24,524.60	26,139.76
Carbonized Khat leaves	17,311.79	15,764.14	18,811.66
Briquettes made from Khat branch with clay soil binder (1:5 ratios)	19,140.46	19,619.68	20,911.81
Briquettes made from Khat leavesclay soil binder (1:5 ratios)	13,849.43	12,611.31	15,049.33
Briquette made from carbonized branch and leavesclay soil binder (1:5 ratios)	16,934.78	16,039.02	17,980.46
Briquette made from carbonized branch with un-carbonized leavesclay soil binder(1:5 ratios)	2,000.62	2,057.24	1,906.96

Table 1.5 Laboratory experimental results ofbriquettes bulk density produced from Khat branch and leaves residue

Samples	Trial 1 (g/cm^3)	Trial 2 (g/cm^3)	Trial 3 (g/cm^3)
Briquettes made from Khat branch with clay soil binder (1:5 ratios)	0.67	0.77	0.75
Briquettes made from Khat leavesclay soil binder (1:5 ratios)	0.69	0.73	0.71
Briquette made from carbonized branch and leavesclay soil binder (1:5 ratios)	0.65	0.76	0.76
Briquette made from carbonized branch with un-carbonized leavesclay soil binder(1:5 ratios)	0.48	0.44	0.53

Archive
Ministry of Water, Irrigation & Electricity

Table 1.6Laboratory experimental results to boil one litter of water; done using Merchaye-stove

Briquette type	Average time taken to boil one litter Water in Minutes	Average time taken to change the water into vapor in (Hour & Minutes)	Average time taken to turn to Ash (Hour & Minutes)
Carbonized Khat branch briquette	23	1hr and 9 Min	2hr and 46 Min
	22	1hr and 12 Min	2hr and 48 Min
	24	1hr and 11 Min	2hr and 50 Min
Carbonized Khat leaves briquette	36	1hr and 19 Min	2hr and 14 Min
	33	1hr and 24 Min	2hr and 10 Min
	37	1hr and 22 Min	2hr and 19 Min

Table 1.7Laboratory experimental resultsof total emission

Emissions	CO_2 (%)	CO (PPM)	O_2 (%)	NO	NO_X
Khat branch residue briquette (Avg)	0.37	728	20.2	2.27	0
Khat leaves residue briquette (Avg)	0.48	831	20.08	7.21	0

Archive
Ministry of Water, Irrigation & Electricity

Khat supply letter

በደቡብ ብሔሮች ፣ ብሔረሰቦች ሕዝቦች
ክልል መንግስት የሀዋሳ ከተማ አስተዳደር
ገቢዎች ባለስልጣን ዋና ቅ/ጽ/ቤት

south nations natonalities & people regional state
Hawassa City Administraton Revenue Authority
Main Branch Office

ቁጥር 1375/7/7/2010
ቀን 12-04-2010

ለሀዋሳ ዩንቨርስቲ ወንዶ ገነት ደ/የተፈ/ሀብት ኮሌጅ
ወንዶ ገነት ፣

ጉዳዩ፡- መረጃን ስለመስጠት ይሆናል፡፡

በርዕሱ እንደተጠቀሰዉ ለጥናት የሚሆን መረጃ ከተቋማችን እንድናመቻች በደብዳቤ በጠየቃችሁን መሠረት የተፈለገውን መረጃ ከዚህ ሸኚ ደብዳቤ ጋር አያይዘን መስጠታችንን እናስታውቃለን፡፡

ከሠላምታ ጋር/

ሙሉጌታ ገ/መድህን አርሳንጎ
Mulgeta G/Medihne Arsango
ሰትድ ልማት ክ/ግ/ግ/መልስ አፈሰር

ግልባጭ
ለሀዋሳ ከተማ ገቢዎች ባለስልጣን
ለአቶ እንዳለ ፍቃዱ
ሀዋሳ ፣

ፖስታ 1640 ስልክ 0462210026
ግብር ለስልጣኔ የሚከፈል ዋጋ ነው፡፡
የታክስ ሙስናን መከላከል ሀገርን ከጠላት መከላከል ነው፡፡

የሀዋሳ ከተማ የጫት ገቢ መረጃ

ዓመተ ምህረት	ኪ.ሎ ግራም	የገቢ መጠን
2005	4,852,508.60	24,262,543.00
2006	5,984,150.20	29,920,751.00
2007	6,863,476.20	34,317,381.00
2008	7,950,227.20	39,751,136.00
2009	6,846,747.20	34,233,736.00
2010ዓ.ም የአምስት ወር	2,389,437.00	11,947,185.00

ማሳሰቢያ፡- የ2010 ዓ.ም መረጃ የአምስት ከሀምሌ/2010ዓ.ም እስከ ህዳር/2010 ዓ.ም ያለው መሆኑን እናሳስባለን፡፡

SNNPRS City Adm Hawassa Revenue Authority Main Branch Office
